# Identifying protein complexes based on node embeddings obtained from protein-protein interaction networks

**DOI:** 10.1186/s12859-018-2364-2

**Published:** 2018-09-21

**Authors:** Xiaoxia Liu, Zhihao Yang, Shengtian Sang, Ziwei Zhou, Lei Wang, Yin Zhang, Hongfei Lin, Jian Wang, Bo Xu

**Affiliations:** 1 College of Computer Science and Technology, Dalian University of Technology, Dalian, 116024 Liaoning People’s Republic of China; 2 Beijing Institute of Health Administration and Medical Information, Beijing, 100850 People’s Republic of China; 3 School of Software Technology, Dalian University of Technology, Dalian, 116024 Liaoning People’s Republic of China

**Keywords:** Node embeddings, Random forest, Supervised learning method, Protein complex detection

## Abstract

**Background:**

Protein complexes are one of the keys to deciphering the behavior of a cell system. During the past decade, most computational approaches used to identify protein complexes have been based on discovering densely connected subgraphs in protein-protein interaction (PPI) networks. However, many true complexes are not dense subgraphs and these approaches show limited performances for detecting protein complexes from PPI networks.

**Results:**

To solve these problems, in this paper we propose a supervised learning method based on network node embeddings which utilizes the informative properties of known complexes to guide the search process for new protein complexes. First, node embeddings are obtained from human protein interaction network. Then the protein interactions are weighted through the similarities between node embeddings. After that, the supervised learning method is used to detect protein complexes. Then the random forest model is used to filter the candidate complexes in order to obtain the final predicted complexes. Experimental results on real human and yeast protein interaction networks show that our method effectively improves the performance for protein complex detection.

**Conclusions:**

We provided a new method for identifying protein complexes from human and yeast protein interaction networks, which has great potential to benefit the field of protein complex detection.

## Background

In recent years, with the development of human genomics and the development of high-throughput techniques, massive protein-protein interaction (PPI) data have been generated. These PPI data have enable to automatically detect protein complexes from PPI networks. During the past decade, most computational approaches used to identify protein complexes have been based on discovering densely connected subgraphs in protein-protein interaction (PPI) networks [1, 2]. However, many true complexes are not dense subgraphs and these approaches show limited performances for detecting protein complexes from PPI networks. At the same time, the unreliable relations in the PPI data also poses a great challenge for protein complex identification [3–5].

Recently, a number of methods have been developed for protein complex identification. Dongen et al. [6] proposed a protein complex discovery algorithm named MCL, which manipulates the adjacency matrix of yeast PPI networks with two operators called expansion and inflation. By iterating these two operators, it will find the clusters that have higher possibility to becoming protein complexes. Bader et al. [7] proposed a protein complex detection algorithm named MCODE which is based on local density to cluster nodes. Zhang et al. [8] introduced a protein complex detection method which measures the likelihood of a subgraph being a real complex based on the number of three node cliques. Liu et al. [9] came up with an algorithm named CMC for protein complex discovery, which uses maximum complete subgraphs as seeds and searches for protein complexes from weighted PPI networks. In this algorithm, the protein interactions are weighted by an iterative scoring weight method called AdjustCD. What’s more, some methods, such as COACH [10] and Core&Peel [11], are proposed for detecting protein complexes based on the core-attachment observation of protein complex. However, most of the above methods are unable to detect overlapping complexes. Recently, Nepuse et al. [12] proposed a method named ClusterONE which utilizes greedy algorithm aggregation for identifying overlapping protein complexes. Some methods, such as Prorank+ [13], also consider the overlapping of protein complexes. In addition, some researchers tried to decrease the negative effects of unreliable PPI data for protein complex detection. For example, Zaki et al. [14] introduced a novel graph mining algorithm (PEWCC) which assesses the reliability of protein interaction by weighting clustering coefficients and removing unreliable edges, then it identifies protein complexes from the new weighted PPI network. All of these algorithms are based on the topological structure of the PPI network and do not utilize the information of known complexes, and these methods have been applied only on the yeast protein interaction networks.

In recent years, some supervised learning methods have been proposed to detect complexes from PPI network by using informative properties of known complexes, including SCI-BN [15], NN [16] and ClusterEPs [17]. These methods usually have three main steps, first they extract features from the known complexes, and then train a supervised classification model or score function to judge whether a subgraph is a true complex, finally use the trained classification model or score function to guide the search process for new protein complexes. However, insufficient extracted features and noise in the PPI data make the classification model imprecise [18]. At the same time, some features are often related to the characteristics of the network, so the features only work on the protein network which has such characteristics, otherwise the performance of complex detection will decrease when the network doesn’t have such characteristics [19]. Therefore, with the increasing amount of data with different characteristics, using traditional features alone fails to further improve the performance of complex detection methods.

However, with the rapid development of deep learning, using self-learned features becomes an alternative way to obtain effective features from networks even with various characteristics. Tang et al. [20] proposed a spectral clustering method based on graph theory in 2011. The basic idea of this method is to use the similarity matrix of the sample data to decompose the features, and then to cluster the obtained eigenvectors, which is only related to sample size rather than sample characteristics. In 2014, Perozzi et al. [21] proposed a method named DeepWalk which learns latent representations of vertices in a network from truncated random walks. This method has achieved a remarkable performance for multi-label network classification task in social networks. In 2015, Tang et al. [22] proposed a method name LINE which learns the *d*-dimensional feature in two phases: *d*/2 breadth-first search simulations and another *d*/2 2 hop distant nodes. In 2016, Grover et al. [23] proposed an algorithm, node2vec, to learn the representations of the nodes in the network. This method creates the ordered sequence simulating breadth-first search and depth-first search approaches. All these above mentioned feature learning approaches aims to learn node embeddings by exploring the structure of networks, and node embedding methods have gained prominence since they produce continuous and low-dimensional features, which obviate the need for task-specific feature engineering and are effective for various task [24]. Thus, those methods enable us to further extract the hidden information from networks, so as to effectively improve the performances of complex detection methods.

Because of above-mentioned reasons, in this paper, we propose a method, NodeEmbed-SLPC-RF, which is based on node embeddings to identify protein complexes on PPI networks. Firstly, it learns the node representations of protein interaction network, then uses the similarities between node representations to quantify the reliability of the PPI interactions in order to filter existing interactions or add new interactions. Secondly, supervised learning method (SLPC [25, 26]) is used to identify candidate protein complexes. Finally, random forest (RF) model is utilized to classify candidate protein complexes and candidate protein complexes with positive labels are outputted as the final predicted complexes. Experimental results show that our method outperforms the state-of-the art methods in detecting protein complexes from PPI networks.

## Methods

We detail our NodeEmbed-SLPC-RF method in this section. Specifically, Node embeddings used in the algorithm are presented, and then SLPC and RF are briefly described, finally NodeEmbed-SLPC-RF algorithm is introduced.

### NodeEmbeded

At present, there are many approaches to generate network node embeddings. Node embeddings are distributed representations for the network nodes, which can be automatically learned based on the network adjacency information and topology structure obtained from the network. Compared with the traditional network structural features, node embedding methods can learn different vector representations for different networks according to their own structures, and thus can quickly mine the characteristics of different networks. And this kind of features are often not expressed by single values, but by dense vectors.

In order to obtain high quality node embeddings, we use node2vec method [23] to automatically get vector representations for all the nodes in the network. Node2vec method learns the low dimensional representations for each nodes and at the same time preserves the structural informations of both the nodes and the network. Particularly, node2vec adapts random walk and aliasing sampling strategy to capture the different local structure of a node. Therefore, the low dimensional representations of the nodes are essentially the feature representations for the nodes.

The node2vec algorithm can be roughly divided into three steps: step 1: obtain transition probability matrix *π* based on return parameter *p* and in-out parameter *q*; step 2: generate node sequences for each node based on *G* and *π* and, *walk* denotes all the node sequences. Specifically, *r* node sequences are generated for each node *v*_*i*_ by using alias sampling strategy and the length of each node sequence is *l*; step 3: use stochastic gradient descent (SGD) strategy to train the model according to *walk* and obtain vectors for each node. Here, the sliding window size for training process is *k*, and the dimension of each vector is *d*. In the algorithm, a graph *G* is searched according to a certain strategy. Particularly, a number of node sequences are generated for each node, and the length of each node sequence is fixed to *l*. The number of sequences is determined by the hyperparameter *r*. And in the algorithm, *k* is the size of the sliding window and *p* determines the probability of traversal from the original path. The larger the *p*, the less likely to return to the same path. Parameter *q* decides the traversal strategy, the larger the *q*, the more likely to use breadth-first search strategy. Node2vec firstly generates the node sequences and all the generated node sequences are used as the contexts of the corresponding nodes. Then the skip-gram architecture [27] is utilized to train the node2vec model and after the training process, the vectors obtained for each node are the learned feature representations for each node. Note that, the time complexity of alias sampling strategy for choosing a node to add into a node sequence is *O*(1).

In this paper, a concept of protein complex vector is proposed. A protein complex is a set of proteins and a protein complex vector is generated by the protein vectors in the set, which is calculated as follows:

1 $$ complex(\phi_{1},\phi_{2},\cdots,\phi_{m})=max\quad \mathbf{Z}(\cdot,j)\quad 0\leq j< d  $$

where *ϕ*_*i*_(*i*=1,2,⋯,*m*) denotes the node embedding of the corresponding protein in the complex, **Z** is the matrix which is composed by *ϕ*_*i*_ in the complex set, *d* denotes the dimension of *ϕ*_*i*_, and **Z**(·,*j*) denotes the *j*-th column of the matrix **Z**.

In addition, as the obtained node embedding vectors not only are the continuous feature representations for nodes in network, but also can reflect the similarities between nodes, we use them to further quantify the reliability of the relations. The vector similarity between two nodes is used to weight the relation between them, and it is defined as follows:

2 $$ similarity(X,Y)=\frac{\sum\limits_{i=1}^{n}x_{i}y_{i}}{\sqrt[]{\sum\limits_{i=1}^{n}x_{i}^{2}}*\sqrt[]{\sum\limits_{i=1}^{n}y_{i}^{2}}}  $$

where *X*=(*x*_1_,*x*_2_,⋯,*x*_*n*_), *Y*=(*y*_1_,*y*_2_,⋯,*y*_*n*_) and *n* is the dimension of the corresponding vector.

### Supervised learning method SLPC

The detail of the supervised learning method (SLPC) used in our work can be found in references [25] and [26]. The SLPC method mainly includes three steps: firstly, a training set, including positive, middle and negative data, is constructed. Secondly, construct the feature vector space for the complexes in the training set from the networks and train the regression model. Specifically, a rich feature set of eleven topological features is constructed for complexes and the regression model is trained with the feature vectors. After that, the proteins whose degrees are greater than the average degree of the network are selected as the initial cliques. Then, the initial cliques are expanded according to the scores obtained by the regression model in order to generate the final cliques which are likely to be the real complexes. The main reason for using supervised learning method in this work is that it can combine the manually selected features with automatic self-learned features to further improve the performance for protein complex detection.

### Random forest

Random forest [28] is a model that uses a large number of sample data to train the decision trees for classification, and the class labels are determined by the output of the decision tree. The main idea of random forest model is as follows. A forest is established in a random way, and the forest is composed of many decision trees, and there is no relation between the trees. When a new sample comes in, each tree makes a decision and a class label is determined if the majority decision trees select this label for the classification task.

Random forest model is tolerant to missing data and unbalanced data as well as it can handle high-dimensional data. During the training process of the random forest model, the number of trees is randomly selected in order to avoid the over-fitting problem. What’s more, it can process the high-dimensional data directly without feature selection process. On the other hand, the importances of each feature can be obtained after training and it can maintain good accuracy even with the missing data and unbalanced data. For protein complex detection task, it is well known that there exist false negative relations in the PPI networks [4, 5], and the number of known standard complexes is quite limited. Therefore, we use random forest model to further filter the candidate complexes based on their features.

### NodeEmbed-SLPC-RF method

**Fig. 1:**
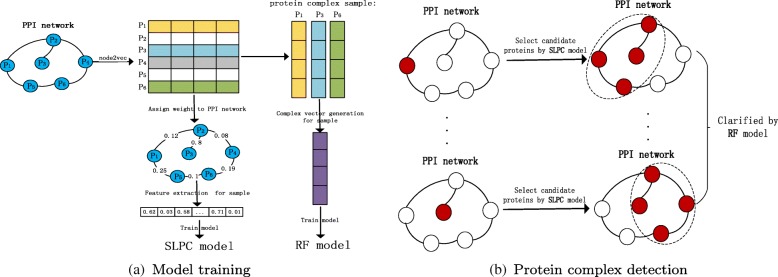
The overall workflow of NodeEmbed-SLPC-RF method. **a** P_1_,P_2_,P_3_,P_4_,P_5_ and P_6_ are the proteins in the PPI network, and P_1_,P_5_ and P_6_ compose a protein complex. **b** The red node in the left network is the seed node, and the nodes in slash circles of the right network is a candidate protein complex discovered by using SLPC model

In this paper, we propose a method named NodeEmbed-SLPC-RF method to detect protein complexes from PPI networks. Figure 1 shows the overall workflow of the NodeEmbed-SLPC-RF method, it can be divided into two main steps. In the first step: the embedding representation of each node is obtained by using node2vec algorithm, then the relations in the PPI network are quantified by using the similarity of node embeddings, and the PPI network is modified based on the reliabilities of the relations. After that, complex vectors of sample complexes are generated according to their corresponding protein vectors for training RF model. At the same time, the SLPC model is trained by using eleven extracted features of sample complexes. In the second step, the trained SLPC model is used to guide the search process for candidate protein complexes from the PPI network. Then the RF model is used to classify the candidate protein complexes, and the protein complexes which are labeled as positive ones are considered to be the final predicted complexes. Specially, there are three categories generated by RF model like SLPC model.

## Results

### Dataset and parameter setting

We conducted the experiments on two different types of PPI networks: Human and Yeast. For human, protein and protein relations were downloaded from the human protein reference database (HPRD) [29], and there were 39,254 interactions and 9678 proteins. For yeast, commonly used DIP network [30] was obtained and there were 17,203 interactions among 4930 proteins in the DIP network. After removing the duplicated and self-linked relations, we obtained 37,060 interactions and 9521 proteins for human and 17,201 interaction and 4928 proteins for yeast. The golden standard of human protein complexes were also downloaded from HPRD, while the golden standard of yeast protein complexes were constructed by combining MIPS [31], Aloy [32], SGD [33] with TAP06 [34]. The total numbers of golden protein complexes are 1514 and 673 and the size of them ranges from 3 to 129, 3 to 359 for human and yeast, respectively.

We evaluated the performance of NodeEmbed-SLPC-RF against SLPC, ClusterONE, MCODE, MCL, CMC, Coach, ProRank+ and PEWCC. We referred to the previous studies [10, 12–14] and used their recommended settings. For ClusterONE, the density threshold, merging threshold, and penalty value of each node were set to 0.6, 0.8 and 2, respectively. For MCODE, MCL, CMC and Coach, we used the recommended settings for unweighted network. For ProRank+ and PEWCC, we used their default settings. In the NodeEmbed-SLPC-RF, the node2vec algorithm is used to learn the feature representations for the nodes on PPI network. In order to embed nodes which have similar structure closer, as suggested by [23], the parameters of node2vec were set as follows: *p*=1,*q*=8,*r*=10,*l*=10,*k*=10. Besides, 1000 trees were used to make decision in the Random forest model.

For the purpose of evaluating the predicted protein complexes, three statistic measures which are widely used in related studies: *precision*, *recall* and *F*−*score* are used as evaluation metrics. Precision is the fraction of the number of the predicted complexes which match at least one golden complex among all predicted complexes. Additionally, recall is the fraction of the golden complexes which match at least one predicted complex over the total number of all golden complexes. The F-score which shows the overall performance is the harmonic mean of precision and recall.

3 $$ F-score=2*\frac{precision*recall}{precision+recall}  $$

Here, the neighborhood affinity score *NA*(*p*,*b*), which is defined as follows, was used to measure the similarity between predicted complex (*p*) with golden standard complex (*q*).

4 $$ NA(p,b)=\frac{|V_{p}\cap V_{q}|^{2}}{|V_{p}|*|V_{q}|}  $$

where |*V*| denotes the set of proteins belong to the corresponding complex. Similar to many previous studies, a predicted complex *p* is regarded to be matched with a golden complex *q* if the *NA*(*p*,*q*) score is not lower than 0.25.

### Experimental results

#### Using complex vectors to classify the candidate complexes

In the experiment, SLPC was used to detect candidate protein complexes from the original network and then RF model was trained to further classify the candidate complexes. Both SLPC and RF are supervised learning methods and the training set for them including the samples of three categories: positive, intermediate and negative samples. Similar to the construction of training set in SLPC [25], the state-of-the-art COACH method [10] was utilized to generate the intermediate complexes since the predicted complexes obtained by COACH have higher possibilities of being true complexes than the negative samples, but lower than the positive ones. Hence, 1175 and 422 complexes predicted by the COACH method for human and yeast were used as the intermediate samples. Therefore, the training sets contain three categories samples, for human: 1521 true complexes from the HPRD database are used as the positive samples, 1175 complexes predicted by the COACH method as the intermediate samples, and 2135 subgraphs obtained by randomly selecting nodes as the negative samples respectively. For yeast: 673 true yeast complexes are used as the positive samples, 422 complexes predicted by the COACH method as the intermediate samples, and 673 subgraphs obtained by randomly selecting nodes as the negative samples respectively. What’s more, the candidate complexes obtained by SLPC were the test data for RF model, and the candidate complexes which were labeled as positive ones were outputted as the final predicted complexes. In the experiment, we used different dimensions of node embedding to generate the complex vector and the experimental results are shown in Table 1. From the Table 1, we can see that using RF model to classify the candidate complexes can decrease the number of predicted complexes but increase the precision and F-score. And the best performance in terms of F-score is obtained when the dimension is set to 64 for both HPRD and DIP networks. The default dimension for the rest of the experiments is 64 for both networks.

**Table 1:** Performance comparison results on HPRD and DIP datasets

Methods	No. of complexes	Precision	Recall	F-score
HPRD				
ClusterONE	789	0.2307	0.1724	0.1973
MCODE	102	0.2059	0.0258	0.0458
MCL	1291	0.1255	0.1704	0.1445
CMC	44	0.3636	0.0178	0.0340
Coach	1762	0.2469	0.3890	0.3021
ProRank+	500	0.2820	0.1625	0.2062
PEWCC	1194	0.2739	0.2299	0.2499
SLPC only	2713	0.3693	0.4901	0.4212
d=32	858	0.7005	0.3785	0.4914
d=64	871	0.7107	0.3983	**0.5105**
d=128	841	0.7099	0.3890	0.5026
d=256	882	0.6961	0.3877	0.4980
d=512	823	0.7096	0.3831	0.4976
d=1024	867	0.7105	0.3970	0.5093
DIP				
ClusterONE	363	0.5069	0.4012	0.4479
MCODE	82	0.0244	0.0030	0.0053
MCL	436	0.3463	0.3952	0.3692
CMC	262	0.4389	0.2912	0.3501
Coach	747	0.4351	0.5156	0.4719
ProRank+	167	0.4731	0.1516	0.2296
PEWCC	666	0.5916	0.3744	0.4586
SLPC only	1061	0.6447	0.4829	0.5522
d=32	719	0.8108	0.4428	0.5728
d=64	710	0.8070	0.4473	**0.5755**
d=128	702	0.8148	0.4368	0.5688
d=256	708	0.8263	0.4413	0.5753
d=512	711	0.8158	0.4413	0.5728
d=1024	691	0.8249	0.4354	0.5699

d denotes the dimension of each vector. No. of complexes denotes the total number of predicted complexes by each method. Bold value denotes the best score corresponding to F-score

We also compared our methods with some supervised methods, namely SCI-BN [15], NN [16] and ClusterEPs [17], on on DIP dataset, which follows the approach used by ClusterEPs. Because the programs of SCI-BN and RM are not available, ClusterEPs compared them based on their published results: therefor, we also compared with their published results. In their experiments, they used MIPS [31] as the known complexes, we tested NodeEmbed-SLPC-RF method under same settings. The results are presented in Table 2. As shown in this table, NodeEmbed-SLPC-RF method has considerably higher scores compared with other supervised methods in terms of F-score.

**Table 2:** Performance comparison results on DIP datasets using the MIPS gold standard

Methods	Precision	Recall	F-score
Ours	0.893	0.581	**0.704**
SPLC only	0.419	0.670	0.514
ClusterEPs	0.649	0.751	0.695
SCI-BN	0.273	0.473	0.346
NN	0.333	0.491	0.397

Bold value denotes the best score corresponding to F-score. Ours denotes the NodeEmbed-SLPC-RF method

**Fig. 2:**
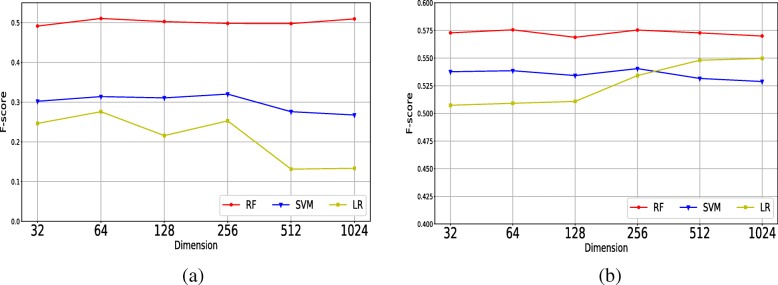
The performance comparison in terms of F-score obtained by SVM, LR and RF with different dimensions on **a** HPRD and **b** DIP

In order to measure the effectiveness of RF model, Support Vector Machine (SVM) and Logistic Regression (LR) which have been proved to be prevalent in classification task [35–37] were used to compare with RF. The experimental results on HRPD are shown in Fig. 2. The y-axis in Fig. 2 denotes the F-score of corresponding positive results obtained by the RF, LR and SVM, respectively. And the x-axis represents different dimensions of node embeddings. It can be seen from the Fig. 2 that the RF model can learn more information from the complex feature vectors and is more effective than LR and SVM in classifying candidate protein complexes in both HPRD and DIP networks.

#### Using node embedding similarities to filter edges from original PPI network

**Fig. 3:**
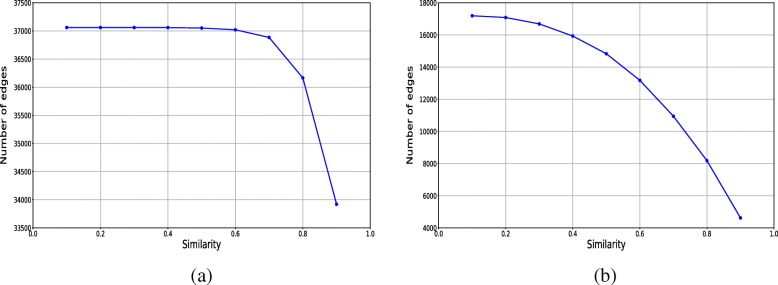
The numbers of edges left after filtering by using different simi-thres on HPRD and DIP. **a** HPRD. **b** DIP

In order to construct more reliable network, the relations in the network were assigned with weights which were calculated by the node embedding cosine similarities, and then some relations with lower weights in the original network were filtered out. In order to find the appropriate similarity threshold (semi-thres) for filtering the edges, we analyzed how many edges could be removed from the original network according to their weights from the original network as shown in Fig. 3. As can be seen from Fig. 3a, when the similarities value increases from 0.8 to 0.9, the number of remaining edges in HPRD decreases greatly. In order to ensure that only noise edges are filtered from the original network, therefore in the experiment, the range of similarity threshold (simi-thres) used in the experiment for HPRD is from 0.8 to 0.9, and the step size is chosen to be 0.01. In addition, from Fig. 3b we can see that when the similarities value increases from 0.65 to 0.75, the number of remaining edges in DIP decreases greatly, even thought the total number of edges in DIP is smaller than HPRD. Therefore, in the experiment, the range of similarity threshold (simi-thres) used in the experiment for DIP is from 0.65 to 0.75, and the step size is chosen to be 0.01. What’s more, the detailed results obtained by using NodeEmbed-SLPC-RF method on the modified network with different simi-thres are shown in Tables 3 and 4.

**Table 3:** Experimental results obtained by using RF to filter the candidate complexes which are predicted from the modified HPRD network by filtering edges with different simi-thres

Simi-thres	No. of edges left	No. of complexes	Precision	Recall	F-score	*Δ*
0.80	36164	999	0.6617	0.4181	0.5124	+0.0912
0.81	36009	999	0.6547	0.4306	0.5195	+0.0983
0.82	35869	1019	0.6487	0.4280	0.5157	+0.0945
0.83	35710	1006	0.6531	0.4293	0.5181	+0.0969
0.84	35523	999	0.6607	0.4326	0.5229	+0.1017
0.85	35311	992	0.6552	0.4359	0.5235	+0.1023
0.86	35117	992	0.6673	0.4326	**0.5249**	**+0.1037**
0.87	34887	979	0.6599	0.4313	0.5216	+0.1004
0.88	34621	975	0.6728	0.4221	0.5187	+0.0975
0.89	34278	950	0.6505	0.4207	0.5110	+0.0898
0.90	33921	943	0.6585	0.4221	0.5144	+0.0932

*Δ* denotes the improvement of F-score compare with using SLPC alone. Bold values denote the best scores corresponding to the specific metric

**Table 4:** Experimental results obtained by using RF to filter the candidate complexes which are predicted from the modified DIP network by filtering edges with different simi-thres

Simi-thres	No. of edges left	No. of complexes	Precision	Recall	F-score	*Δ*
0.65	12167	653	0.8760	0.4413	0.5869	+0.0347
0.66	11941	667	0.8726	0.4428	**0.5875**	**+0.0353**
0.67	11683	652	0.8712	0.4368	0.5819	+0.0297
0.68	11423	634	0.8801	0.4324	0.5799	+0.0277
0.69	11174	617	0.8995	0.4294	0.5813	+0.0291
0.70	10946	612	0.9020	0.4235	0.5764	+0.0242
0.71	10673	610	0.8918	0.4235	0.5743	+0.0221
0.72	10410	616	0.8929	0.4235	0.5745	+0.0223
0.73	10184	622	0.8939	0.4264	0.5774	+0.0252
0.74	9907	608	0.8947	0.4160	0.5680	+0.0158
0.75	9633	594	0.9091	0.4190	0.5736	+0.0214

*Δ* denotes the improvement of F-score compare with using SLPC alone. Bold values denote the best scores corresponding to the specific metric

#### Using node embedding similarities to augment the original network

Since the feature vector representations for each node in the network were obtained by node2vec and the similarities between vector representations might reflect the connectivity between two protein nodes, for each target node, a new relation was generated by determining which one had the highest similarity with the target node. Then some of the new relations were integrated into the original network if the similarity between two nodes was larger than a certain threshold. Finally, the NodeEmbed-SLPC-RF algorithm was used to identify candidate complexes from the integrated network.

**Fig. 4:**
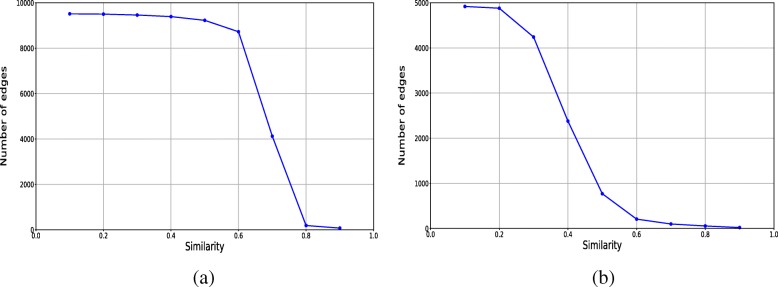
The numbers of edges added by using different simi-thres on HPRD and DIP. **a** HPRD. **b** DIP

In order to find the appropriate simi-thres to add new relations, the similarities of all the new relations were analyzed and Fig. 4 shows the distribution of the similarities of the new relations for HPRD and DIP. As can be seen from Fig. 4a, when the similarity increases from 0.65 to 0.75, the number of added edges for HPRD significantly decreases. In order to ensure the number and the quality of new added edges, the similarity threshold (simi-thres) used in the experiment for HPRD ranges from 0.65 to 0.75, and the step size is set to be 0.01. As we can see from Fig. 4b, when the similarity increases from 0.35 to 0.45, the number of added edges for DIP significantly decreases, although the total number of added edges is smaller than HPRD. The similarity threshold (simi-thres) used in the experiment for DIP ranges from 0.35 to 0.45 in order to ensure the number of added edges, and the step size is set to be 0.01. Specifically, after integrating new edges into original networks according to the different simi-thres, SLPC algorithm is used to identify candidate complexes, and then RF model is used to classify the candidate complexes in terms of their complex feature vectors to obtain the final predicted complexes. The detailed experimental results are shown in Tables 5 and 6.

**Table 5:** Experimental results obtained by using RF to filter the candidate complexes which are predicted from the modified HPRD network by adding edges with different simi-thres

Simi-thres	No. of added edges	No. of complexes	Precision	Recall	F-score	*Δ*
0.65	7889	717	0.6137	0.2893	0.3932	-0.0280
0.66	7572	824	0.6104	0.3454	0.4412	+0.0200
0.67	7174	829	0.6164	0.3487	0.4455	+0.0243
0.68	6531	940	0.6266	0.3983	0.4870	+0.0658
0.69	5546	952	0.6313	0.4003	0.4899	+0.0687
0.70	4121	1030	0.6544	0.4168	0.5092	+0.0880
0.71	2522	1021	0.6513	0.4148	0.5068	+0.0856
0.72	1390	1028	0.6566	0.4207	**0.5129**	**+0.0917**
0.73	850	1015	0.6611	0.4155	0.5102	+0.0890
0.74	583	1024	0.6563	0.4188	0.5113	+0.0901
0.75	447	1017	0.6608	0.4168	0.5111	+0.0899

*Δ* denotes the improvement of F-score compare with using SLPC alone. Bold values denote the best scores corresponding to the specific metric

**Table 6:** Experimental results obtained by using RF to filter the candidate complexes which are predicted from the modified DIP network by adding edges with different simi-thres

Simi-thres	No. of added edges	No. of complexes	Precision	Recall	F-score	*Δ*
0.35	3351	702	0.8305	0.4428	0.5776	+0.0254
0.36	3153	707	0.8317	0.4458	0.5804	+0.0282
0.37	2979	698	0.8295	0.4398	0.5748	+0.0226
0.38	2784	696	0.8290	0.4473	**0.5810**	**+0.0288**
0.39	2586	691	0.8234	0.4413	0.5746	+0.0224
0.40	2378	677	0.8198	0.4339	0.5674	+0.0152
0.41	2196	685	0.8161	0.4354	0.5678	+0.0156
0.42	2019	698	0.8095	0.4383	0.5687	+0.0165
0.43	1831	689	0.8084	0.4339	0.5647	+0.0125
0.44	1634	703	0.8108	0.4413	0.5715	+0.0193
0.45	1473	710	0.8056	0.4413	0.5702	+0.0180

*Δ* denotes the improvement of F-score compare with using SLPC alone. Bold values denote the best scores corresponding to the specific metric

#### Link prediction by using different methods

The node2vec algorithm is used to obtain the node embeddings in our method, since it can learn rich feature representations for nodes in a network. We conducted link prediction experiments in order to validate the effectiveness of node2vec algorithm. Link prediction problem aims to predict whether a link exists between two nodes in a network. It is well known that nodes with common neighbors tend to form future links [38], so we compared node2vec with two methods which are based on the common neighbors. One is the AdjustCD algorithm [9] and the other is PE-measure [14]. Given a pair of nodes *u* and *v*, the AdjustCD score is calculated as:

5 $$ AdjustCD(u,v)=\frac{2|N_{u}\cap N_{v}|}{max(|N_{u}|,|N_{avg}|)+max(|N_{v}|,|N_{avg}|)}  $$

where *N*_*u*_ and *N*_*v*_ are the numbers of the neighbors of each node, and $N_{avg}=\frac {\sum _{x\in V}|N_{x}|}{N}$ is the average number of neighbors in the network and N is the total number of nodes in the network. PE-measure is an iterative method for calculating the score between node *u* and *v*. Suppose that matrix **P**(*k*) is the score matrix in *k* iteration, then the score between *u* and *v* is the element *p*(*k*)_*uv*_ of matrix **P**(*k*) which can be calculated as:

6 $$ p(k)_{uv}=1-\prod(1-p(k-1)_{ul}\cdot p(k-1)_{vl})  $$

where it takes the product by all *l*: (*u*,*l*)∈*E*, (*v*,*l*)∈*E*. In the experiment, the number of iterations *k* was set to 2 as suggested by [14].

For node2vec, cosine similarity is used to calculate the score of two nodes based on their obtained embeddings. In the test, we first hide a *T* percentage of edges randomly sampled from the network, while ensuring that the remaining network remains connected. These "hidden" edges are considered as the ground truth, then we would like to predict these edges. In this test, mean ranking and Hits@N are adopted to evaluate the effectiveness of link prediction, and for each pair of nodes *u* and *v*, another 100 nodes that are not connected to *u* are selected as candidate nodes. Considering the fact that the predicted top-ranked results are more important in practice, we measure the performance of different methods in terms of the top-ranked results, i.e, the mean ranking of true edges, and the proportion of true edges ranked in the top *N* results. Usually, it is regarded as more effective if the method can rank more true edges in the top portions. In the test, 10% percentage of edge were removed from the network. We summarize our results for link prediction in Table 7. The dimension of node2vec is 64 and the random denotes using random vectors with dimension equals to 64. From the Table 7 we can see that node2vec outperforms in terms of all metrics in all the datasets except that AdjustCD has better performance in terms of Hits@10 on HPRD. We also tested the effects of different dimensions for link prediction, Table 8 shows the results with different dimensions, and the performance is the best when dimension equals to 64 in both HPRD and DIP. To sum up, the results demonstrate the efficacy of node2vec on link prediction in two real-world PPI networks, which suggests that node2vec is able to effectively learn the proper feature representations for the nodes in the PPI networks.

**Table 7:** Comparison results for link prediction on HPRD and DIP

Method	Mean ranking	Hits@1	Hits@10	Hits@50
HPRD				
random	52.87	2	7.8	47.8
node2vec	**24.79**	**29.8**	53.4	**78.4**
PE	35.53	25.64	52.14	70.09
AdjustCD	35.07	23.93	**60.68**	68.38
DIP				
random	49.01	2.8	10.8	51.4
node2vec	**10.50**	**59**	**80.4**	**91.4**
PE	30.73	3.8	29.8	75.4
AdjustCD	29.03	8.8	37.8	77.4

Bold values denote the best scores corresponding to the specific metric. The value of each column in terms of Hit@N with different *N* is the percentage of true edges ranked in top *N*

**Table 8:** Comparison results for link prediction with different dimensions by using node2vec on HPRD and DIP

Dimension	Mean ranking	Hits@1	Hits@10	Hits@50
HPRD				
d=32	25.37	28.6	51.2	76.4
d=64	**24.79**	**29.8**	**53.4**	**78.4**
d=128	25.83	27.6	52.8	76.2
d=256	27.62	26.6	49	74
d=512	27.74	27.8	47.2	75.4
d=1024	27.22	25.8	50	74.2
DIP				
d=32	12.76	54	74.4	89.2
d=64	**10.50**	**59**	**80.4**	91.4
d=128	11.45	59	80.2	90
d=256	10.77	57	79	**91.6**
d=512	11.19	54.4	79	90.6
d=1024	10.65	52	77	91.2

Bold values denote the best scores corresponding to the specific metric. The value of each column in terms of Hit@N with different *N* is the percentage of true edges ranked in top *N*

#### Using different strategies to generate complex vectors

As described in the method section, the complex vector is generated based on its corresponding node embeddings of proteins in the complex. In order to evaluate how the generation strategy of complex vector affects the performance of NodeEmbed-SLPC-RF, we conducted experiments with three different complex vector generation strategies on both HPRD and DIP networks. The Table 9 shows the effectives of different vector generation strategies with the dimension sets to 64. As we can see from the table, using max value of each column of the matrix **Z**, which is composed by the corresponding node embeddings in the complex, to generate complex vector obtains better performance than others on both HPRD and DIP, the reason may be that max operation gathers the global important features from all the node embeddings of proteins in the specific protein complex.

**Table 9:** Performance comparison using different vector generation strategies on HPRD and DIP datasets

Methods	No. of complexes	Precision	Recall	F-score
HPRD				
Max	871	0.7107	0.3983	**0.5105**
Min	854	0.7037	0.3824	0.4956
Average	937	0.6126	0.354	0.4487
DIP				
Max	710	0.8070	0.4473	**0.5755**
Min	701	0.8160	0.4368	0.5690
Average	698	0.8181	0.4354	0.5683

Bold value denotes the best score corresponding to F-score. Max denotes selecting the max value of each column of the matrix **Z** which is composed by the corresponding node embeddings in the complex. Min denotes selecting the min value of each column of the matrix **Z**. Average denotes getting the average value of each column of the matrix **Z**

## Discussion

In the previous section, complex vector is generated by its corresponding node embeddings and the complex vectors are considered as features for RF model to further classify the candidate complexes. From the Table 1 we can see that using RF model to further classify candidate complexes could improve the performance of protein complex detection in terms of F-score, however the improvement on DIP is relatively slight. For example, when the dimension of vector is set to be 64, the F-score could improve 8.93% compared with that of using SLPC alone on HPRD network, however the F-score only improves 2.33% compared with that of using SLPC alone on DIP network. In order to measure the effectiveness of RF, we also compare it with SVM and LR, and the comparison result is shown in Fig. 2. It can be seen from the figure that using classifier does not necessarily improve the experimental results. Compared with RF model, SVM and LR model are less effective, especially on HPRD network. This shows that RF can learn effective information of complex feature vectors, while SVM and LR can learn relatively limited information. The reason may be that they have different ways for learning features. In addition, the decision function of SVM is determined by a small number of support vectors, and the overlap between the complexes may interfere with the its decision function thus leading to the poor performance of SVM. What’s more, the LR model is based on a linear function which normally can’t achieve promising result when it encounters linearly non-separable problem [38].

As mentioned in section of filtering edges, the original PPI network was reconstructed by filtering lower reliable edges based on the node embedding similarities between nodes, then SLPC was used to identify candidate complexes from the modified PPI network, and finally RF model was utilized to classify the candidate complexes based on their complex feature vectors in order to obtain the final predicted complexes. It can be seen from Fig. 3, the similarities of the majority relations in the original PPI network are greater than 0.8 and 0.65 on HPRD and DIP respectively, which indicates that the entire network is closely related for HPRD than DIP. However, there are still some relations which have lower similarities. By filtering the relations which have lower connectivity can help to delete the unreliable relations, so as to effectively improve the performance of complex detection methods. As can be seen from Tables 3 and 4, using NodeEmbed-SLPC-RF method on the modified networks can greatly improve the experimental results. The highest F-score is obtained on the modified network with the simi-thres equals to 0.86 for HPRD, which is about 10.37% higher than that on the original network with using SLPC alone. In addition, the highest F-score on the modified DIP network is with the simi-thres equals to 0.66, but it is only 3.53% higher than that on the original network with using SLPC alone. However, the results show that filtering the relations according to their similarities with proper simi-thres can help to improve the performance for protein complex detection.

As mentioned in the section of augmenting networks, we calculated the similarities between all the node pairs using their node embeddings and then added new relations whose similarities were greater than a threshold to the original PPI networks. Then the candidate complexes were predicted by the SLPC algorithm, and finally the candidate complexes were further classified by RF model to obtain the final predicted complexes. It can be seen from Fig. 4 that the number of the addable edges varies when the simi-thres ranges from 0.65 to 0.75 and 0.35 to 0.45 on HPRD and DIP respectively, which indicates that the similarity scores of most relations are greater than 0.65 and 0.35 on HPRD and DIP respectively. In order to obtain a more effective threshold of similarity for adding new relations into the original networks, we tested the performance of NodeEmbed-SLPC-RF method with the threshold of similarity ranging from 0.65 to 0.75 and 0.35 to 0.45 on HPRD and DIP respectively. The experimental results are shown in Tables 5 and 6. As can be seen from the Table 5, when the similarity threshold is 0.72, the highest F-score can be obtained, which is 9.17% higher than that on the original network with SLPC alone. In addition, from the Table 6 we can see that the best F-score is obtained with simi-thres equals to 0.36, but the improvement is slight compared with the F-score obtained by SLPC alone on the original network. All in all, these results show that adding reliable relations according to their similarity scores can effectively improve the performance of our model.

In addition, we also conducted a experiment which was designed by filtering edges in accordance with the idea of the section of filtering lower reliable edges and then adding new relations in accordance with the idea of the section of augment networks. In order to find the appropriate threshold, for HRPD we first fixed the filtering simi-thres to be 0.86 as using this semi-thres our model can reach best F-score as shown in Table 3, then the adding simi-thres varied from 0.65 to 0.75 to find the appropriate threshold for adding new relations. Table 10 shows the detailed results by using different simi-thres on HPRD. It can be seen from the Table 10, when the filtering simi-thres is set to 0.86 while the adding simi-thres is set to 0.74, the model obtains the best performance in terms of F-score. Also, we fixed the adding simi-thres to 0.72 according to the best result in terms of F-score in Table 4, and then the filtering score varied from 0.80 to 0.90. The results is shown in Table 10. Furthermore, for DIP we first fixed the filtering simi-thres to be 0.66 as using this semi-thres our model can reach best F-score as shown in Table 4, then the adding simi-thres varied from 0.35 to 0.45. Table 11 shows the detailed results by using different simi-thres on DIP. It can be seen from the Table 11, when the filtering simi-thres is set to 0.66 while the adding simi-thres is set to 0.40, the model obtains the best performance in terms of F-score on DIP. Similar to HPRD, we then fixed the added simi-thres for DIP with 0.38, and the filtering simi-thres ranged from 0.65 to 0.75. The detailed results can be found in Table 11. It can be seen from Table 11 that using node embedding similarities to filter the relations first and then adding new relations into the network can slightly improve the performance of NodeEmbed-SLPC-RF for protein complex detection. For example, the F-score of our method on DIP network can be increased by about one point compared with that of only filtering relations from the original DIP network, which demonstrates that node embedding similarity can reflect the connectivity between nodes and further proves that adding new reliable relations based on their similarities can be an effective way to improve the performance of detecting protein complexes from PPI network.

**Table 10:** Experimental results obtained by using RF to filter the candidate complexes which are predicted from the modified HPRD network by filtering edges first and then adding edges with different simi-thres

Simi-thres	No. of complexes	Precision	Recall	F-score	*Δ*
fixing filtering sime-thres to 0.86					
0.86_0.65	1018	0.6234	0.3547	0.4521	+0.0309
0.86_0.66	1137	0.5638	0.4075	0.4731	+0.0519
0.86_0.68	1151	0.5656	0.4095	0.4751	+0.0539
0.86_0.67	874	0.6545	0.4135	0.5068	+0.0856
0.86_0.69	868	0.6544	0.4135	0.5068	+0.0856
0.86_0.70	872	0.6514	0.4148	0.5068	+0.0856
0.86_0.71	872	0.6560	0.4135	0.5072	+0.0860
0.86_0.72	952	0.6702	0.4293	0.5234	+0.1022
0.86_0.73	967	0.6660	0.4267	0.5201	+0.0989
0.86_0.74	981	0.6758	0.4293	**0.5251**	**+0.1039**
0.86_0.75	978	0.6708	0.4300	0.5240	+0.1028
fixing adding sime-thres to 0.72					
0.80_0.72	903	0.6755	0.4062	0.5073	+0.0861
0.81_0.72	897	0.6778	0.4188	0.5177	+0.0965
0.82_0.72	975	0.5908	0.3791	0.4619	+0.0407
0.83_0.72	905	0.6862	0.4221	0.5226	+0.1014
0.84_0.72	888	0.6926	0.4194	0.5224	+0.1012
0.85_0.72	907	0.6880	0.4221	0.5232	+0.1020
0.86_0.72	952	0.6702	0.4293	**0.5234**	**+0.1022**
0.87_0.72	871	0.6820	0.4161	0.5169	+0.0957
0.88_0.72	890	0.6685	0.4102	0.5084	+0.0872
0.89_0.72	853	0.6694	0.4089	0.5076	+0.0864
0.90_0.72	856	0.6600	0.4055	0.5024	+0.0812

*Δ* denotes the improvement of F-score compare with using SLPC alone. Bold values denote the best scores corresponding to the specific metric

**Table 11:** Experimental results obtained by using RF to filter the candidate complexes which are predicted from the modified DIP network by filtering edges with simi-thres 0.66 first and then adding edges with different simi-thres

Simi-thres	No. of complexes	Precision	Recall	F-score	*Δ*
fixing filtering sime-thres to 0.66					
0.66_0.35	665	0.8797	0.4428	0.5891	+0.0369
0.66_0.36	659	0.8786	0.4398	0.5862	+0.0340
0.66_0.37	667	0.8741	0.4398	0.5852	+0.0330
0.66_0.38	660	0.8758	0.4443	0.5895	+0.0373
0.66_0.39	673	0.8678	0.4413	0.5851	+0.0329
0.66_0.40	669	0.8789	0.4443	**0.5902**	**+0.0380**
0.66_0.41	669	0.8714	0.4428	0.5872	+0.0350
0.66_0.42	667	0.8786	0.4428	0.5888	+0.0366
0.66_0.43	672	0.8705	0.4398	0.5844	+0.0322
0.66_0.44	667	0.8741	0.4413	0.5865	+0.0343
0.66_0.45	667	0.8771	0.4398	0.5859	+0.0337
fixing adding sime-thres to 0.38					
0.65_0.38	594	0.8064	0.4086	0.5424	-0.0098
0.66_0.38	660	0.8758	0.4443	**0.5895**	**+0.0373**
0.67_0.38	707	0.7765	0.4250	0.5493	-0.0029
0.68_0.38	681	0.7797	0.4190	0.5451	-0.0071
0.69_0.38	687	0.7729	0.4160	0.5409	-0.0113
0.70_0.38	676	0.7678	0.4086	0.5334	-0.0188
0.71_0.38	664	0.7636	0.4071	0.5311	-0.0211
0.72_0.38	678	0.7478	0.4071	0.5272	-0.0250
0.73_0.38	677	0.7518	0.4042	0.5257	-0.0265
0.74_0.38	678	0.7552	0.4012	0.5240	-0.0282
0.75_0.38	655	0.7588	0.3923	0.5172	-0.0350

*Δ* denotes the improvement of F-score compare with using SLPC alone. Bold values denote the best scores corresponding to the specific metric

## Conclusion

In this paper, we propose a protein complex detection method which is based on node embeddings, and the results demonstrate that our method can effectively improve the performance for detecting protein complexes from PPI network. Specifically, compared with using SLPC alone, when using RF model to classify the candidate complexes generated by SLPC based on their complex feature vectors and the candidate complexes labeled as positive by RF model were considered as the final predicted complexes, the performance in terms of F-score can be improved up to 8.93% and 2.33% on HPRD and DIP, receptively. In addition, When the original relations were filtered based on the similarity scores of node embeddings and the candidate complexes were further classified according to their complex vectors, the performance in terms of F-score can be increased up to 10.37% and 3.53% on HPRD and DIP respectively compared with using SLPC alone. The results indicate that the performance of protein complex detection methods could be improved by using node embeddings obtained by node2vec to measure the reliability of exiting relations in the PPI networks. What’s more, when adding new relations according to their similarity scores and using complex vectors to filter the candidate complexes, the performance in terms of F-score can be increased by up to 9.17% and 2.88% on HPRD and DIP respectively compared with using SLPC algorithm alone. To sum up, the experiment results demonstrate the effectiveness of using node embeddings and complex vectors for detecting protein complexes from PPI networks. In future work, we will further explore how to combine node embeddings with biological resources for predicting complexes from PPI network.
